# The Effect of Curing Conditions on the Service Life of 3D Printed Concrete Formwork

**DOI:** 10.3390/ma16216972

**Published:** 2023-10-30

**Authors:** Michiel Bekaert, Kim Van Tittelboom, Geert De Schutter

**Affiliations:** Magnel-Vandepitte Laboratory for Structural Engineering and Building Materials, Department of Structural Engineering and Building Materials, Faculty of Engineering and Architecture, Ghent University, Technologiepark Zwijnaarde 60, B-9052 Ghent, Belgium; kim.vantittelboom@ugent.be (K.V.T.); geert.deschutter@ugent.be (G.D.S.)

**Keywords:** 3D printed concrete, service life, durability

## Abstract

Complex concrete elements are typically produced with lost formwork made out of timber or plastic. After usage, these timber or plastic panels are disposed of. This makes complex lost formwork a polluting and high-cost-inducing aspect of concrete construction. A possible solution for this problem could be 3D printing of concrete. This high degree of freedom construction process could easily be used to produce complex formwork. As the formwork stays in place, it has a function during and after the hardening of the inner concrete. Before hardening, the formwork keeps the fresh concrete in place. After hardening, the printed formwork takes the function of a concrete cover. The concrete cover protects the steel reinforcement against aggressive environmental substances such as chlorides and carbon dioxide. To properly execute this function, the printed material and the transition between printed material and inner concrete need to perform at least as well as the inner material. This experimental research investigates the usability of a 3D printed concrete mixture as a concrete cover in a combined concrete structure. The effect of the curing condition as well as two different surface finishing techniques of the printed formwork are taken into account. The effect of the different parameters is compared based on existing service life models. Results indicate that proper curing of the printed formwork is of key importance in order to obtain significant resistance against carbonation- and chloride-induced corrosion. Adjusting the nozzle with side trowels improves the resistance of the printed material against chloride intrusion and carbonation but has only a limited effect on the service life extension.

## 1. Introduction

Three-dimensional printed formwork is intended to remain in its place when the infill concrete is hardened. A that point, its function as a formwork has been fulfilled. However, instead of becoming death weight, the 3D printed formwork could take a new function: concrete cover. Within this paper, the possibility of 3D printed formwork to act as a protective layer for concrete reinforcement is investigated. The paper consists of an experimental and numerical part. In the experimental part, the effect of the curing conditions of the printed formwork and possible improvement techniques are investigated. The experimental results are used to calculate the service life of the intended formwork to obtain a better understanding of the possibility of its new function.

## 2. Durability

The number of applications where 3D printed concrete is used has significantly increased in the last couple of years. Walls, columns, and stairs are only a couple of elements that are currently being printed [1,2]. However, these printed concrete elements are hollow and do not contain any form of traditional reinforcement. The applications for these types of unreinforced elements are limited to low loadbearing structures with a high aesthetic value. As a solution to reinforce these printed structures, the hollow silhouettes could be used as formwork for traditional concrete [3]. In this way, the large degree of freedom of printed concrete and the knowledge of traditional concrete can be combined. In these elements, reinforcement can be easily placed in the cast material. However, considering the design, the reinforcement’s exact position depends on whether the printed concrete can be treated as concrete cover. 

### 2.1. Design of the Concrete Cover

**Figure 1:**
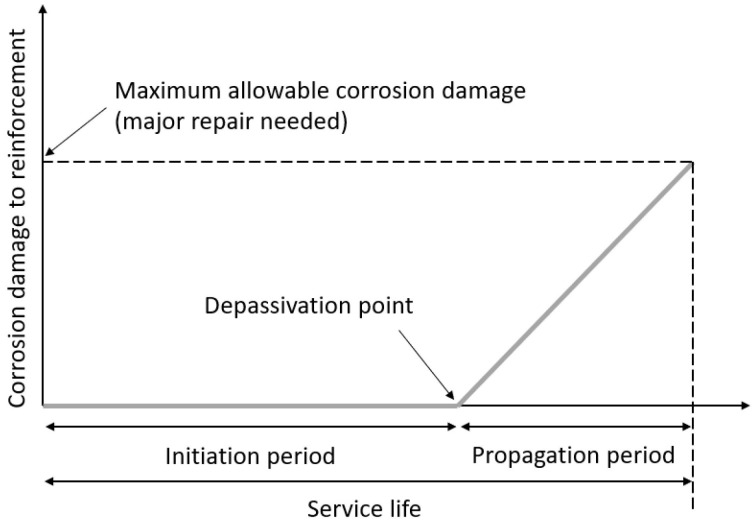
The service life of a reinforced concrete structure.

The concrete cover protects the reinforcement of the concrete against aggressive substances such as carbon dioxide and chlorides. The concrete cover is a porous material resulting in the fact that after a certain period, aggressive substances can reach the reinforcement and depassivation can start. The period for the substances to reach the rebars is called the initiation period [4]. When the substances reach the reinforcement (depassivation point), the rebars will start to corrode. From this point onwards, the reinforcement will start to corrode and cracks and spalling of the concrete cover is observed. This period is called the propagation period [5]. Each of these two time periods is dependent on several parameters. The initiation and propagation period together form the service life of the reinforced structure, as shown in Figure 1 [6]. The service life means the assumed period in which no major repairs are necessary. However, as the initiation period is typically significantly longer than the propagation period, the point of depassivation (start of the corrosion) can be considered as a proxy for the service life [7,8]. 

To predict the service life of reinforced concrete, the International Federation of Structural Concrete (Fib) provided model code 34, the code for service life design [7]. The code describes a full probabilistic design method to calculate the service life of concrete elements subjected to chloride- and carbonation-induced corrosion. The model is retrieved from the projects DuraCrete and DARTS [7]. When designing structures resistant to chloride ingress, an additional Fib bulletin is provided with additional information about benchmark values and distributions of the incorporated parameters [9]. As the model code considers numerous variables (e.g., concrete composition, binder type, curing regime) all data is related to cast concrete and not to printed concrete. Therefore, the effect of printing on the resistance factor should be studied further. 

### 2.2. Effect of Curing on Durability

One of the main advantages of 3D concrete printing is that no formwork is needed. However, a concrete formwork does not only support the freshly cast concrete, but it also protects the concrete from drying out at an early age. It is highly warned that an early removal will lead to (plastic) cracking of the concrete [10]. Nevertheless, as mixing water evaporates due to the lack of formwork, the relative humidity within the concrete starts to decrease. When the relative humidity in the concrete becomes too low, the cement hydration will be affected too. It has been reported that the hydration of cement paste containing only Portland cement (PC) stops when the internal relative humidity drops below 75–80% [11,12]. As the hydration hampers, the porosity of the concrete is increased in comparison to properly hydrated concrete [13]. This effect increases drastically when using supplementary cementitious materials (SCM) such as ground granulated blast-furnace slag (GGBS). GGBS is a latent hydraulic SCM, which means its hydration only comes after the start of the cement hydration and in the presence of enough water [14]. This implies that substituting OPC with GGBS in a concrete mix design leads to a reduction in the early-age hydration kinetics [15]. This slower hydration process results in lower compressive strength during the early stages [16,17], a higher carbonation coefficient [15], and increased porosity [15]. Over time, more hydration products will begin to form and fill the present voids, leading to a decrease in porosity [18]. However, a problem occurs when a significant amount of water has evaporated during the early stages. In such a scenario, the hydration may slow down, which leads to an insufficient amount of hydrates to fill the pores [14,18]. As a result, the concrete will have an increased quantity of coarse pores, which will reduce the durability of the matrix. As the hydration rate of GGBS-incorporated concretes is lower than that of pure PC–concrete at an early age, the curing conditions become more important at an early age [19]. Therefore, the effect of the curing conditions on the resistance against carbonation, chloride ingress, and frost-thaw has been an important research topic. 

As previously mentioned, the durability is affected by the curing condition of the concrete [20]. The increase in porosity eases the penetration of carbon dioxide and chlorides into the concrete structure. However, the mix composition must be taken into account as well. In the case of pure PC-concrete, there is still a significant amount of Portlandite (Ca(OH)_2_) present. This means that the alkalinity of the concrete remains high, which benefits the carbonation resistance. However, in the case of GGBS blended types of cement, this alkalinity is reduced compared to a pure PC mix [19]. As a result, the carbonation, which is already influenced by the curing conditions, will also be affected by the quantity of incorporated GGBS [19]. Nevertheless, it has been shown that implementing GGBS into the concrete improves its resistance to chloride ingress. Therefore, the curing effect and the mixture composition will play a significant role in the durability performance. 

### 2.3. Curing of 3D Printed Concrete

Inherent to the 3D printing technology is the lack of formwork during the production of the elements. Despite this being an opportunity to reduce the required formwork material, the concrete characteristics can be negatively affected. Formwork not only holds the concrete in place during the fluid state, but also protects the concrete from early drying and improves the curing too. The lack of cover affects the mechanical properties and durability aspects of the printed material. Ma et al. [21] investigated the effect of the drying conditions (standard conditions: RH > 95% and dry conditions: RH = 60 ± 5%) on the mechanical properties of 3D printed samples. It was observed that flexural and compressive strength decreased by 20–30% when curing the specimens in dry conditions rather than in a standard condition. Federowicz et al. [22] analyzed the effect of the curing method (dry curing, external curing, and internal curing) on the shrinkage behavior. External curing in the form of a plastic sheet cover was found to be the most effective in reducing the shrinkage. However, fully covering printed structures is not always feasible. Superabsorbent polymers (SAPs) can be implemented to improve the curing of the printed elements. The SAPs can gradually release water, leading to internal curing and keeping the internal RH high at an early age. Van Der Putten et al. [23] implemented SAPs in printed concrete and found a reduction in autogenous shrinkage. Nevertheless, Moelich et al. [24] argued that adding SAP could change the rheological properties of the printed material. Therefore, Moelich et al. [24] proposed the use of curing agents, which would not influence the rheological properties, to reduce the pore water evaporation and hence the shrinkage. The researchers reported that curing agents effectively reduced the shrinkage and related cracking. The reduction was attributed to the lower rate of pore water evaporation. At the same time, it was suggested that this would also improve the durability performance of the material. In another study, Moelich et al. [25] investigated the effect of evaporation rates during curing (LAB: 0.02 kg/m^2^/h and SITE: 0.36 kg/m^2^/h) and the curing in a water tank on the durability performance of 3D printed concrete. The oxygen permeability decreased by water curing the samples. Samples cured in the SITE condition showed 23% more permeability than those cured in LAB condition. Similar, the porosity of the water cured specimens was significantly lower than the non-water cured specimens. However, samples cured under SITE condition obtained a significantly lower porosity than those cured under LAB conditions. The authors suggested that the porosity may be related to the magnitude of early age shrinkage with a higher magnitude reducing the porosity. 

### 2.4. Effect of Printing on Durability

The curing process of the printed concrete is of importance, but so is the placing method. The resistance of the concrete mix is highly dependent on its porosity. A large porosity facilitates the ingress of aggressive substances. The porosity of concrete can be improved intentionally (by lowering the water-to-cement ratio [26], adding secondary cementitious materials [19]) or deteriorated unintentionally (wall effect [27] or improper curing [19]). In general, printing is believed to have a negative effect on the porosity of the concrete matrix. Kruger et al. [28] observed an increase in porosity when comparing printed (7.9% porosity) to cast samples (6.8% porosity). Additionally, the influence of the interlayer is suggested to be negligible in case of a small time gap. However, Rahul et al. [29] mentioned that it is mainly the interlayer between two printed layers that has a negative effect, as it was observed that the bulk material had a lower porosity than the interlayer zone. Since the porosity is location-dependent for printed concrete (due to the interlayer), its resistance to chloride and carbonation also varies [14]. This observation was made by Van Der Putten et al. [30]. The ingress front of chlorides tested on printed specimens increased with the time gap between the layers, resulting in a non-uniform distribution of chloride penetration. However, the front was uniform for both cast and printed specimens if the time gap was equal to zero. In the latter case, the ingress depth of the printed samples was higher than for the cast samples. 

When printed concrete is used as formwork material, an additional interface is formed between the cast and printed material. Sanchez et al. [31] investigated the microstructure in the cast-print interface based on thin section analysis and SEM-EDX. Within the contact surface of the printed concrete, an increased degree of hydration of the printed concrete was observed. The authors pointed out that this increase could be related to the water sorption of the cast concrete into the formwork or the carbonation of the specimens during curing of the printed formwork. However, no specific conclusion was made. 

The durability of printed formwork has been investigated only once in real-life structures. Wangler et al. [32] observed the damage on 3D printed columns filled with conventional concrete after two years of exposure to a high alpine environment. The authors concluded that the design plays a significant role in the durability performance of the printed formwork. Adequate matchmaking between the cast and printed material is needed to obtain a proper result. 

In most of the previously reported studies, only the effect of printing on the durability performance of the concrete mixture was investigated. However, the early exposure of the printed element to the air and the considered curing condition can have a significant effect. These aspects have not yet been investigated. Therefore, in this experimental study, the influence of the curing condition on the durability performance of printed concrete is investigated. In addition, an attempt to improve the durability of printed concrete is carried out by adding side trowels to the nozzle.

## 3. Materials and Methods

Within the scope of this paper, two different test sequences were performed. In the first test sequence, the effect of the curing conditions on the durability performance of printed elements was investigated on three different mixtures. The performance of the sole printed concrete is of interest as this can indicate the best curing technique of the formwork. In the second sequence, the durability performance was tested on properly cured formwork in combination with cast concrete.

### 3.1. Materials and Mix Composition 

One 3D printed concrete mixture (REF) was used throughout the whole study. In sequence 1, two additional different printed mixtures were tested (M1, M2). The printed mixtures were designed as provided by Mohan et al. [33]. The printable concrete contains two types of binders: a ground granulated blast-furnaced slag (GGBS) and Portland cement CEM I 52.5 N. Seasand 0/1 is used as a small aggregate. The mixtures have a water-to-cement ratio of 0.35. To improve the flowability and buildability of the mixtures, a superplasticizer (MasterGlenium^®^ 51con 35%, Master builders Solutions Belgium nv, Ham, Belgium) and a viscosity modifying agent (Tylose MOT 60000 YP4, SE Tylose GmbH & Co. KG, Wiesbaden, Germany) were added. The mix compositions and properties can be found in Table 1. 

The self-compacting concrete (SCC), which will be cast into the printed formwork, is designed by Desnerck et al. [34] and meets the requirements of concrete used in a sea environment exposed to rain and frost (EE2). The mixture consists out of Portland cement CEM I 52.5 N and limestone filler. Sand 0/4 and two types of gravel (2/8 and 8/16) are used as aggregates. To improve the flowability, a superplasticizer (MasterGlenium^®^ 51 con 35%, Master builders Solutions Belgium nv, Ham, Belgium) is implemented. The mix composition is given in Table 2.

**Figure 2:**
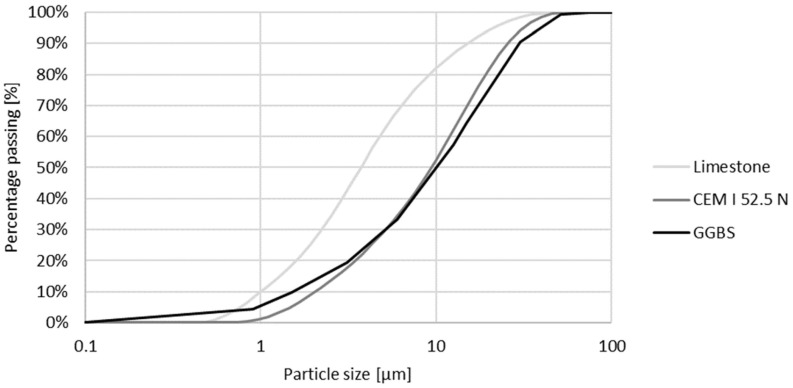
Cumulative particle size distribution of the binders and filler.

**Figure 3:**
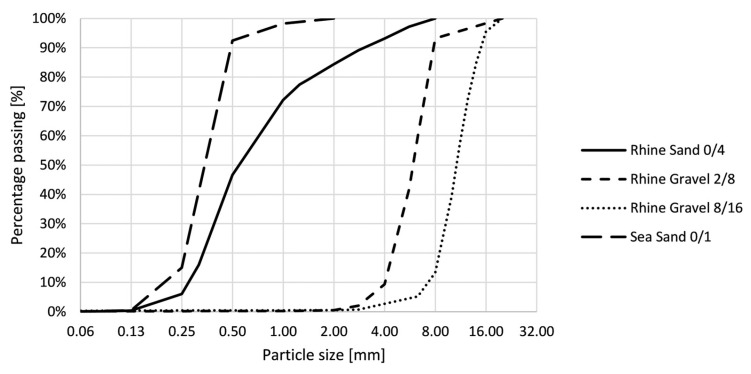
Cumulative strain size distribution of the aggregates.

The cumulative grainsize distribution of the binders and fillers and the cumulative particle size distribution of the aggregates are shown respectively in Figure 2 and Figure 3. 

### 3.2. Printing Procedures

#### 3.2.1. Sequence 1: The Effect of the Formwork Curing Conditions 

**Figure 4:**
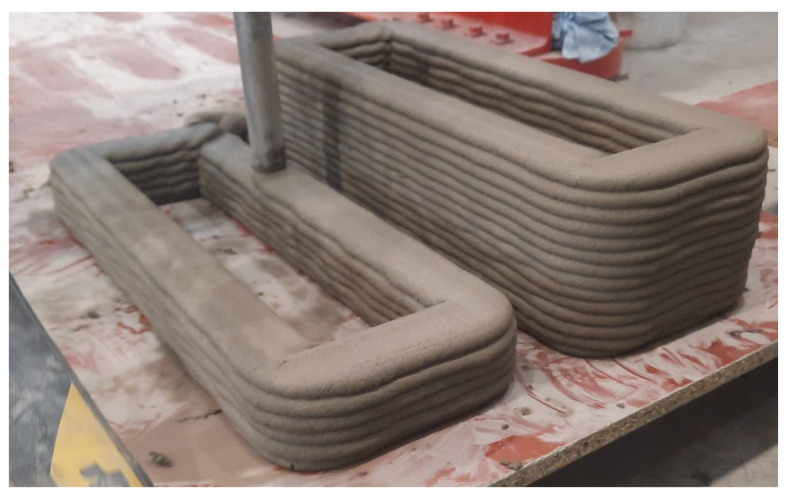
Rectangular specimens printed for sequence 1.

Rectangular elements (500 mm × 150 mm) were continuously printed with three different mixtures (M1, M2 and REF), as shown in Figure 4. Specimens had a layer height of 10 mm and contain 6 or 14 layers. The specimens had a nominal layer width of 40 or 60 mm. For each mixture, two rectangles were printed. The maximum time gap was limited to 12 s and a printing speed of 105 mm/s was used. A circular nozzle with diameter 25 mm and free outflow was used to print the specimens.

After production, specimens were either immediately covered with foil and cured in a climatized room (RH > 95%; T: 20 ± 1 °C) or left exposed to the air and cured in a climatized room (RH = 60 ± 5%; T: 20 ± 2 °C). All specimens were cured for 28 days before they were wet-sawn into specimens with a length of 50 mm. The samples were oven-dried (40 °C) up to a constant mass (<0.1 m% difference after 24 h) before further test preparations.

#### 3.2.2. Sequence 2: The Performance of Properly Cured Elements

Linear wall elements were printed (printing speed: 105 mm/s) with a circular nozzle (diameter 25 mm) with a free outflow or compressed outflow (addition of side trowels to the nozzle with a layer width of 40 mm). The specimens had a layer thickness of 10 mm and a nominal width of 40 mm. Depending on the performed test, the specimens had a total height of 100 mm or 130 mm and a length of 400 mm. After printing, the specimens were immediately covered with foil to prevent evaporation.

After 28 days of curing of the printed concrete (RH = 60 ± 5%; T: 20 ± 2 °C), the printed wall elements (400 mm × 100 mm × layer width) were placed in steel molds and SCC was cast next to the formwork. The new combined specimens are demolded the day after and are wrapped in aluminum foil to only expose the printed side to the air of the climatized room (RH = 60 ± 5%; T: 20 ± 2 °C). In this way, the cast concrete is not in contact with the open air. The specimens were then cured for another 35 days in the climatized room (RH = 60 ± 5%; T: 20 ± 2 °C).

Traditional cast specimens were prepared by casting the printable material in molds (400 mm × 100 mm × 100 mm). The concrete was compacted by a vibrating table. The specimens were demolded after one day and three of the four sides were wrapped in foil. The traditional specimens were immediately cured for 64 days in the climatized room (RH = 60 ± 5%; T: 20 ± 2 °C). The specimens were cured 64 days, which aligns with the curing time of the 3D printed part of the combined elements. This results in a better comparison of both production methods. 

### 3.3. Testing Methods: Effects Based on Experimental Methods

Several tests have been performed for the different sequences. Within these sequences, different variables were taken into account. In Table 3 and Table 4, an overview of all performed tests together with the variables are shown. For each test, the specific testing dates and the quantity of samples tested for each combination are given. 

#### 3.3.1. Evaporation of Water

The difference in the evaporation rate between the two curing conditions (RH > 95%; T: 20 ± 1 °C) and (RH = 60%; T: 20 ± 2 °C) was investigated with mixture REF. Nine specimens were made of one layer (30 mm × 30 mm × 160 mm). Three specimens were exposed to both curing conditions and three control specimens were made, which were fully covered with foil. At different periods, the mass of the samples was measured. The test was performed up to 96 h (4 days). The mass loss is assumed to be related to the evaporated water. The results are reported as the evaporation rate (1):

(1) $$i=\frac{m_{j}-m_{j+1}}{A*∆t}$$

where i is the evaporation rate [kg/m^2^/h], $m_{j}$ and $m_{j+∆j}$ are the mass of the specimen at measurement j and j+1, A is the projected surface exposed to the air (only the side and top surface of the specimens) [m^2^], and ∆t is the time between two measurements [hours]. Based on the results, an estimation can be made of the water quantity left in the specimens (2):

(2) $$w\%=\frac{w_{j}}{w_{total}}$$

where w% is the cumulative water loss [%], $w_{j}$ is the cumulative water loss at measurement j [g], and $w_{total}$ is the theoretical quantity of water immediately after mixing [g]. 

#### 3.3.2. Porosity Based on Vacuum Saturation

**Figure 5:**
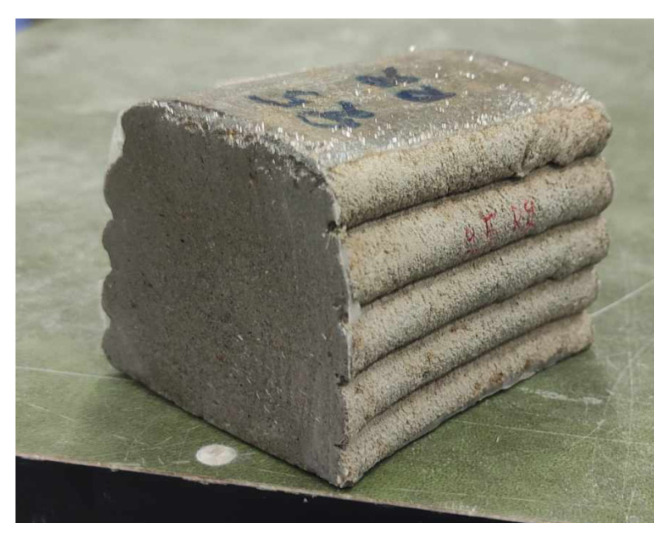
Coated specimen for porosity test.

The vacuum saturation was performed according to NBN EN 1936 [35], but only on sequence 1 and sequence 2 cured specimens. The specimens were coated at the top and bottom to only expose the sides to the water. The specimens were coated with one layer of epoxy (Episol^®^ DesignTop SF, Koramic Chemicals, Wommelgem, Belgium). This can be seen in Figure 5. 

The specimens were placed in a vacuum chamber on line supports. The air pressure was decreased to 2 ± 0.7 kPa. After 2 h, water was gradually inserted into the chamber without increasing the pressure, until the specimens were fully submerged. After another hour, the pressure was slowly increased up to atmospheric pressure. The mass increase was measured for the first time 24 h after submersion had started. The specimens were deemed fully saturated when the mass did not significantly increase (<0.1 m%) after 24 h. At this point, the hydrostatic weight and the saturated mass were obtained. Based on the results, the open porosity $p_{open}$ (3) was obtained.

(3) $$p_{open}=\frac{m_{sat}-m_{d}}{m_{sat}-m_{h}}*100$$

where $m_{d}$ is the oven-dry mass of the specimen [g], $m_{sat}$ is the fully saturated mass of the specimen [g], $m_{h}$ is the mass of the specimen immersed in water [g], and $\rho_{water}$ is the density of the water [998 kg/m^3^].

#### 3.3.3. Water Absorption 

The samples were coated after drying with one layer of epoxy at the top and the bottom side. The nominal dimensions of the exposed surface were measured before testing. Additionally, the mass of the coating and the dry sample was determined by measuring the specimen before and after coating in dry condition. Before testing, the specimens were assumed to be dry as no mass loss was measured after 24 h of drying in an oven at a temperature of 40 °C. The test was performed by placing the specimens in a box on line supports, exposing the side to the water. Water (20 °C) was added until the water level was 5 ± 2 mm above the top of the line supports. On top of the container, a lid was placed to prevent evaporation. The mass of the samples was measured after excess water was removed from the exposed surface by a wet cloth. The mass was obtained at 1, 3, 7, 15, 30, and 60 min and every hour up to 6 h, 24 h, and 144 h after water contact. The results of the test are calculated based on Equation (4).

(4) $$w(t)=\frac{m_{wet}(t)-m_{coating}-m_{dry}}{A_{projected}}$$

where w(t) is the cumulative water intrusion at a time *t* [g/mm^2^], $m_{wet}$ is the wet mass of the sample at a time *t* [g], $m_{coating}$ is the mass of the coating [g], $m_{dry}$ is the oven dry mass [g], and $A_{projected}$ is the projected area of the exposed surface [mm^2^]. The test was performed for the specimens in test sequences 1. 

#### 3.3.4. Accelerated Carbonation (AC)

Sequence 1: The effect of the formwork curing conditions

Before starting, three samples were split to obtain the initial and natural carbonation of the preparation period. The other samples were placed in a carbonation chamber (RH: 65%; T: 21 °C; CO_2_: 1%). Due to a high carbonation rate observed after one week of exposure, the measuring periods were chosen as: 7, 14, 21, and 28 days. On these predefined periods, the carbonation front was obtained by splitting the samples and spraying phenolphthalein on the surface. A picture was taken of every tested surface. The carbonation front of all the samples was analyzed with ImageJ^®^ v.1.54f. For each surface, the carbonation depth was measured every 2 mm. The average of all these depths is the carbonation depth of one sample. For each exposure time, six samples were tested.

Sequence 2: The performance of properly cured elements

**Figure 6:**
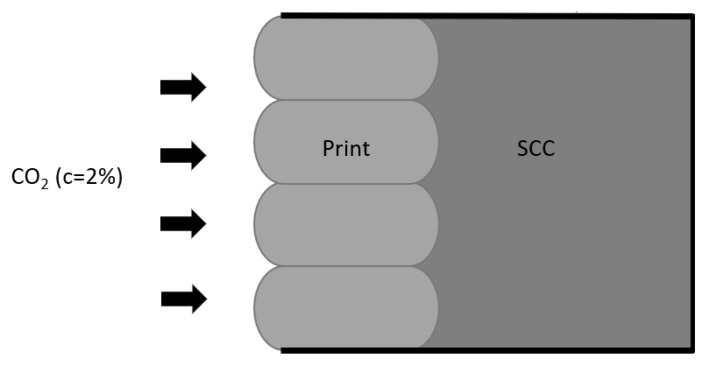
Setup accelerated carbonation of test sequence 2.

After 64 days of curing of the combined samples, the specimens were placed in the CO_2_-chamber (RH: 65%; T: 21 °C; CO_2_: 2%) without drying in the oven. Before testing, a reference measurement was taken to determine the natural carbonation that occurred during curing. Only the printed side or one side of the cast prisms (a molded side) is exposed during testing as the other sides were sealed by aluminum foil, as indicated in Figure 6. To measure the carbonation ingress, slices of a minimum of 20 mm are taken from each prism after 0, 7, 14, 28, and 56 days of exposure. Phenolphthalein is sprayed on the surface of the slices to see the carbonation front. On each split slice, at the middle of the bulges, the ingress is measured. For the printed specimens, the ingress is measured at the bulges as the ingress front is uniform due to the limited time gap between the two layers. 

#### 3.3.5. Rapid Chloride Migration (RCM)

Sequence 1: The effect of the formwork curing conditions

After 25 days of curing of the printed specimens, 3 concrete cores (Ø100 mm × height 50 mm) were drilled. For all specimens, the bulk material was tested, which means that the curved surfaces were removed until no bulges were visible anymore. Afterwards, the specimens were flattened. Three specimens were tested for each test series (n = 3).

Sequence 2: The performance of a combined element

For all tested specimens, 3 cylinders (Ø100 mm × height 50 mm) are drilled after 63 days of curing. For the cast samples, cores are drilled out of cubes while for the printed specimens the cores are drilled out of the printed wall elements. The bulk material of printed elements is tested by removing the curved outer part up to the point that no bulges are visible anymore. When investigating the effect of the surface finishing, the curved outer layer is kept and exposed to the chloride solution. Three samples were tested for each series (n = 3).

Testing procedure of RCM

**Figure 7:**
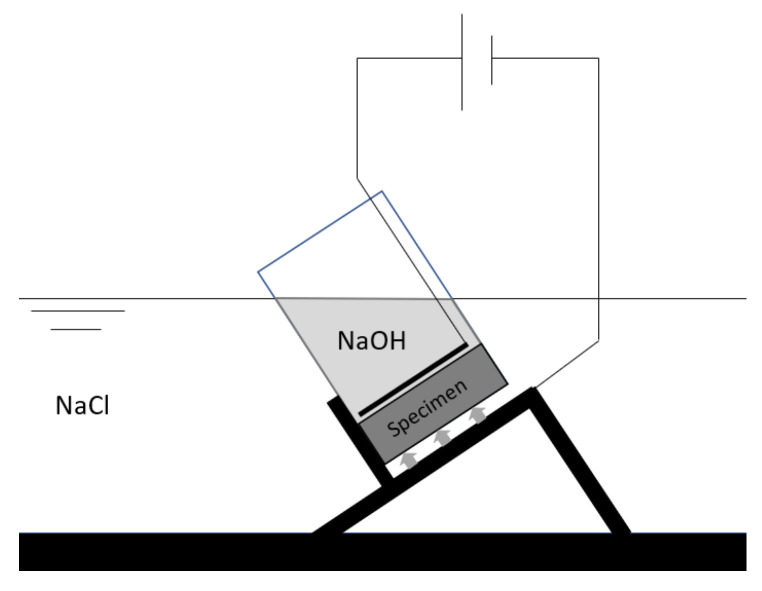
Rapid chloride migration test setup.

The test was performed according to NT Build 492 [36]. Specimens are exposed to a vacuum for 4 h. One hour before ending the vacuum, a saturated Ca(OH)_2_ solution is added to submerge the specimens. Twenty-four hours after the vacuum exposure started, the specimens are placed in the setup to test the chloride ingress, as shown in Figure 7. The water level of the NaOH (0.3 M) and the NaCl-solution (10%) is equal, so no additional liquid pressure is present during testing. A voltage is applied over the specimens based on the initial current in the system. After 24 h, the specimens are taken out of the setup and split. By spraying silver nitrate (0.1 M) on the surface, the ingress of chlorides can be measured in 7 different places on each side. Based on equation 5 of NT Build 492 [36], the chloride migration coefficient is determined.

(5) $$D_{nssm}=\frac{0.0239\left(273+T\right)*L}{\left(U-2\right)t}(x_{d}-0.0238\sqrt{\frac{\left(273+T\right)Lx_{d}}{U-2}})$$

where *D_nssm_* is the non-steady state migration coefficient [×10^−12^ m^2^/s], *U* is the applied voltage [V], *T* is the average value of the initial and final temperatures in the anolyte solution [°C], *L* is the thickness of the specimen [mm], *x_d_* is the average value of the penetration depths [mm], and *t* is the test duration [hour]. 

### 3.4. Numerical Method: Effects Based on Service Life Analyses 

To properly investigate the influences of the different variables on the durability of concrete structures, the effects are compared with each other based on the corrosion initiation period. Within this paper, the ending of this period (the depassivation point) is indicated as the service life. This makes it also possible to assess the effect of the performed enhancement technique, independent of the testing method. 

The service life indicates in this case the period needed for aggressive substances to reach the reinforcement. The length of this initiation period can be estimated based on both chloride ingress and carbonation tests. These experimental values can be combined with benchmark values in a limit state function. By solving these limit state functions, the failure probability for a specified concrete cover thickness after a certain period can be found. Once the failure probability exceeds a certain threshold value, the service life is deemed to end. For the service limit state described in Fib 34 [7], a maximum value of 10% for the failure probability is allowed. This corresponds to a reliability index of 1.3. In this study, the service life is estimated with the equations reported in [7,9]. The failure probability of the cover is calculated with COMREL^®^ v.2023.1 based on the First Order Reliability Method.

#### 3.4.1. Specification Regarding the Concrete Cover Design

To predict the service life of the tested 3D printed formwork, some assumptions have to be made to design the concrete structure.

1. The designed structure is located in the exposure class XS1. The metrological data is taken from the Belgian coastal area;
2. The structure is designed for a service life of 50 years. This is the default design service life for buildings and common structures;
3. The used experimental values are the bulk properties of the printed concrete.

Taking into account these parameters, the required concrete cover could be estimated for carbonation and chloride-induced corrosion. Moreover, a possibility exists that the service life of a predetermined printed layer thickness can be estimated. For example, the prescribed concrete cover according to Eurocode 2 for the given assumptions is 45 mm. However, it should be noted that current models are only validated for cast concrete and have never been evaluated for printed concrete. 

#### 3.4.2. Carbonation-Induced Corrosion 

In the Fib 34 model code [7], the full probabilistic design method for carbonation-induced corrosion in an uncracked element is discussed. The limit state function (Equation (6)) evaluates the concrete cover *d* to the carbonation depth *x_c_(t)* at a certain point of time *t*.

(6) $$g\left(a,x_{c}(t)\right)=d-x_{c}(t)$$

where *d* is the concrete cover [mm] and $x_{c}(t)$ is the carbonation depth [mm] at time *t* [years]. The calculation of the carbonation depth $x_{c}(t)$ can be written as Equation (7).

(7) $$x_{c}\left(t\right)=\sqrt{2*k_{e}*k_{c}*R_{NAC,0}^{-1}*C_{s}}*\sqrt{t}*W(t)$$

where $k_{e}$ is the environmental function [-], $k_{c}$ is the execution transfer parameter [-], $R_{NAC,0}^{-1}$ is the inverse carbonation resistance, $C_{s}$ is the CO_2_ concentration [kg/m^2^], *t* time [years] and the weather function [-]. The environmental function $k_{e}$ takes into account the influence of the relative humidity on the diffusion coefficient and the carbonation resistance during the exposure time.

(8) $$k_{e}={\left(\frac{1-{{RH}_{real}}^{f_{c}}}{1-{{RH}_{ref}}^{f_{c}}}\right)}^{g_{c}}$$

where ${RH}_{real}$ is the relative humidity of the carbonated layer [-], ${RH}_{ref}$ is the reference relative humidity [-], and $f_{c}$ and $g_{c}$ are the exponents [-]. The execution transfer parameter $k_{c}$ takes into account the effect of curing on the effective carbonation resistance.

(9) $${k_{c}=\left(\frac{t_{c}}{7}\right)}^{b_{c}}$$

where $t_{c}$ is the period of curing [days] and $b_{c}$ is the exponent of regression [-]. The inverse carbonation resistance $R_{NAC,0}^{-1}$ can be obtained by the following equation.

(10) $${R_{NAC,0}^{-1}=k}_{t}*{R_{Acc,0}}^{-1}+\varepsilon_{t}$$

where ${R_{Acc,0}}^{-1}$ is the inverse effective carbonation resistance of dry concrete determined on specimens with accelerated carbonation test [(mm^2^/year)/(kg/m^2^)] and $k_{t}$ is the regression parameter considering the test influence and $\varepsilon_{t}$ is the error term considering inaccuracy when using the accelerated carbonation method [(mm^2^/year)/(kg/m^2^)]. The inverse carbonation resistance of the accelerated test can be calculated based on Equation (11).

(11) $${R_{Acc,0}}^{-1}=(\frac{x_{c}}{\tau }{)}^{2}$$

where $x_{c}$ is the measured carbonation depth after 28 days [mm] and τ is a time constant for the described test conditions [(mm^2^/year)/(kg/m^2^)]^0.5^. 

However, one must keep mind that the latter equation is based on the specific test performed as described in Fib 34 [7]. Changes in the accelerated carbonation test (ACC) will influence the obtained inverse carbonation resistance. However, within this testing campaign, some adaptations had to be made. Therefore, transformations and adaptations were needed:

1. In the prescribed test method, a carbonation concentration of 2% is requested. However, this concentration created a too-high carbonation rate for sequence 1. Therefore, the concentration was lowered to 1%. According to Audenaert [37], the carbonation coefficients are related to the exposed CO_2_-concentration, as shown in Equation (12).
  (12) $$\frac{R_{acc,1\%}}{R_{acc,2\%}}=\frac{\sqrt{c_{acc,1\%}}}{\sqrt{c_{acc,2\%}}}$$
  where $R_{acc,1\%}$ and $R_{acc,2\%}$ are the carbonation rates under respectively 1% and 2% CO_2_ concentration and $c_{acc,1\%}$ and $c_{acc,2\%}$ are the corresponding concentrations of CO_2_. Similar conversions have been performed by Van den Heede et al. [38] and Sisomphon and Franke [39]. However, they pointed out that such a conversion is allowed with a maximum CO_2_ concentration of 3% [38];
2. The ingress of the carbonation front after 28 days (x) was calculated based on the obtained carbonation rate after regression instead of the data obtained after 28 days of exposure. It is believed that this would reduce the measurement error. The regression equation was of the form:
  (13) $$x=R_{acc}*\sqrt{t}$$
  where *t* is the exposure time [days], $R_{acc}$ is the carbonation rate, and *x* is the carbonation ingress.

The last parameter in the equation is the weather function, $W\left(t\right)$. This parameter takes into account the weather conditions, which can lead to wetting of the concrete surface.

(14) $$W\left(t\right)={\left(\frac{t_{0}}{t}\right)}^{\frac{{\left(p_{sr}\;\cdot \;TOW\right)}^{b_{w}}}{2}}$$

where $t_{0}$ is the reference time [years], $p_{sr}$ is the probability of driving rain, TOW is the time of wetness (or the percentage days with at least 2.5 mm rainfall per year) [-], and $b_{w}$ is the exponent of regression [-]. 

All data (in combination with their statistical distribution) to estimate the service life can be found in Table 5. The distributions related to the parameters are based on the reported values in Fib 34 [7]. The weather-related data is retrieved from the Royal Meteorological Institute (RMI), see Refs. [40,41]. In [41], data from two weather stations in the Belgian coastal area (Koksijde and Middelkerke) between respectively 1952 and 2016 and between 1955 and 2016 were analyzed to obtain the related statistical distribution. 

#### 3.4.3. Chloride-Induced Corrosion

The durability of reinforced concrete structures exposed to chlorides is explained in Fib bulletin 76 [9]. The service life *t* of the concrete elements ends at the moment a critical chloride content $C_{crit}$ [m%/c] is reached at the reinforcement depth *d* [m]. This leads to the limit state function of Equation (15).

(15) $$g\left(C_{crit},C\left(x=d,t\right)\right)=C_{crit}-C\left(x,t\right)$$

The quantity of chlorides at depth *d* after a certain service time *t* $\left(C\left(x=d,t\right)\right)$ can be described by Equation (16).

(16) $$C\left(x,t\right)=C_{0}+\left(C_{s}-C_{0}\right)*(1-\mathrm{erf}\left(\frac{x-∆x}{2*\sqrt{D_{app}(t)*t}}\right))$$

where $C_{0}$ is the initial chloride content [m%/c], $C_{s}$ is the chloride content resulting from the prevailing exposure environment at depth ∆x [m%/c], ∆x is the depth of the convection zone [m], x is the depth with the corresponding chloride content $C\left(x,t\right)$ [m], *t* time [years], $D_{app}(t)$ is the apparent chloride diffusion coefficient [m^2^/s] and erf is the error function.

The apparent chloride diffusion coefficient $D_{app}(t)$ is known to decrease with increasing exposure time. Therefore, $D_{app}(t)$ can be rewritten depending on the age of the exposed structure. Two methods exist to take aging into account: one based on a short-term laboratory diffusion test (Approach A) and one based on a RCM test (Approach B). The type of approach is dependent on the estimation of the lifetime. As the RCM test does not produce the same degree of chloride binding and the long-term interaction with the saline solution, the estimated $D_{app}(t)$ based approach B will be unfavourable and result in an underestimation of the service life. Approach A can better approximate this interaction, which results in a better service life estimation. However, proper testing to estimate the effect of aging on the chloride diffusion takes a long time (a least 2 years), which was not possible in this study. Therefore, the $D_{app}(t)$ was determined according to approach B.

(17) $$D_{app}\left(t\right)=k_{e}\cdot D_{RCM}\left(t_{0}\right)\cdot {\left(\frac{t_{0}}{t}\right)}^{a}$$

where $k_{e}$ is the environmental variable [-], $D_{RCM}\left(t_{0}\right)$ is the chloride migration coefficient at the reference point in time [m^2^/years], $t_{0}$ is the reference point in time [years], and a is the aging exponent according to Approach B. 

The environmental variable takes into account the ambient temperature. As chloride transport is a thermodynamic process, this cannot be overseen. The effect of the temperature on the diffusion process can be written based on the Arrhenius equation.

(18) $$k_{e}=\exp\left(b_{e}\left(\frac{1}{T_{ref}}-\frac{1}{T_{real}}\right)\right)$$

where $b_{e}$ is the temperature coefficient [K], $T_{ref}$ is the reference temperature [K], and $T_{real}$ is the temperature of the structural element or the ambient air [K].

The data used to estimate the service life by chloride-induced corrosion is given in Table 6. The distribution of the model parameters and values (except with the RCM related test data) are retrieved from [9]. The weather-related data are retrieved from [40,41].

## 4. Effects Based on the Experimental Results

### 4.1. Evaporation

**Figure 8:**
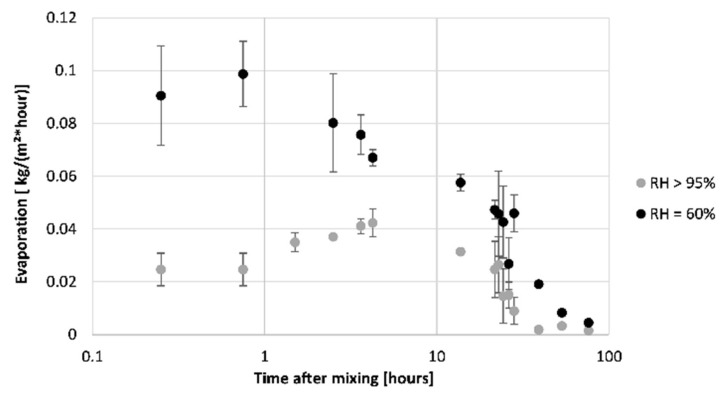
Evaporation rate of mixing water from the printed samples for different curing conditions; n = 3.

Figure 8 shows the moisture evaporation of REF, which are concrete samples in various curing conditions immediately exposed to air after mixing. Concrete exposed to a RH = 60% has a higher initial evaporation rate than specimens cured in RH > 95%. A maximum evaporation rate of 0.1 kg/(m^2^·h) was observed, which is significantly lower than the threshold value of ACI (1 kg/m^2^·h) [42]. Exceeding the latter threshold value is stated to result in a high risk of plastic cracking of the concrete. 

According to Bakshi et al. [43], two different phases in drying exist based on the saturation degree of concrete. These phases can be observed in the evaporation rate. In the first stage (stage I), continuous evaporation indicates that the concrete is fully saturated, and a continuous liquid phase exists. A uniform vapor pressure (which is equal to the saturated one) is present within the concrete. In the case of RH > 95%, the evaporation rate is similar within the first 24 h. For the RH = 60% condition, this is only the case up to 2 h. As drying continues, the specimens obtain an insular saturation state (stage II). This means that the liquid phase becomes discontinuous within the concrete [44]. In this stage, diffusion of water vapor dominates moisture transport. The evaporation rate will decrease, and the vapor pressure will deviate from the saturated one. Evaporation will continue until the ambient vapor pressure or a hydraulic potential balance is reached. For the tested specimens, evaporation changes drastically at 24 h for both curing conditions. Nevertheless, for RH = 60%, the transition from stage I to stage II starts after 2 h of drying, while for RH > 95% this is after 24 h. This indicates that the specimens at RH = 60% become unsaturated after 2 h and the vapor pressure starts to drop locally towards the vapor pressure of the curing room. This means that the RH within the concrete will decrease towards ambient RH. 

**Figure 9:**
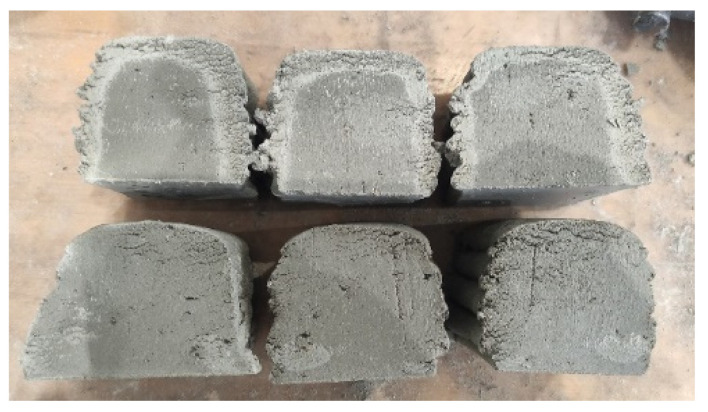
Visual observation of the drying front formed within the first 24 h of curing in REF specimens. The top specimens were exposed to air (T: 20 °C; RH = 60%) and the bottom specimens were covered with foil.

One must keep in mind that the decrease in RH is not uniform within the printed concrete, but will change gradually throughout the depth of the specimens. As a result, a drying front will be formed within the printed material. This can be observed in Figure 9. The specimens exposed to air have bulk material that is darker grey than the outside of the printed specimens, indicating a higher moisture content. For the specimens that were covered, a uniform color is observed, which indicates more uniform drying. 

**Figure 10:**
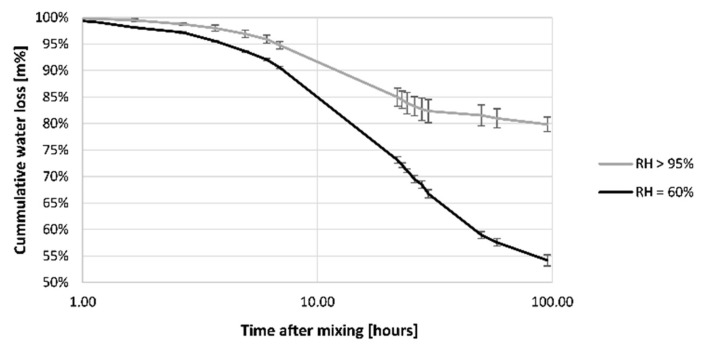
Remaining mixing water in REF concrete under the different curing conditions; n = 3.

The immediate exposure to air leads to a large reduction in water content within the first hours. Additionally, over the first days a significant amount of water evaporates, which can be seen in Figure 10. In the case of RH > 95%, 20% of the mixing water was evaporated after 4 days, while for RH = 60% it was 45%. As GGBS is latent hydraulic, the high moisture loss could lead to the hampering of the hydration reaction and an increase in porosity. In combination with the observation of Figure 9, this is expected to happen within the drying affected zone of the printed concrete. 

### 4.2. Open Porosity Based on Vacuum Saturation

**Figure 11:**
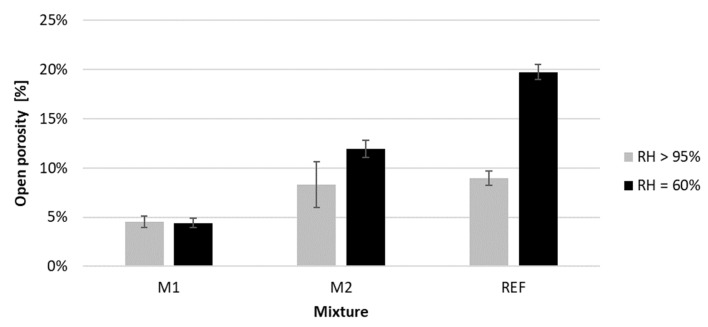
Open porosity of the printed concrete cured under RH > 95% and RH = 60% for 28 days; n = 3.

Figure 11 shows the obtained open porosity of printed samples from the three tested mixtures after 28 days of curing under RH > 95% and RH = 60%. The results indicate a higher open porosity of the two mixtures with GGBS (REF and M2) incorporated than those without GGBS (M1) cured under a high relative humidity curing condition (One-way Anova test, *p*-value: 0.02). The porosity of the M2 and REF mix is not significantly different under this curing condition (Independent sample *t*-test, *p*-value: 0.61). However, when curing at a lower RH, there is a significant difference between the open porosity of all mixtures (One-way Anova test, *p*-value < 0.001).

First of all, the porosity of the M1 mixture is not significantly affected by the curing condition, which is not the case for the GGBS-incorporated mixtures. The porosity of M2 and REF increased respectively by 44% and 120% when immediately exposing the concrete to drying (RH = 60%). The increase in porosity is mainly the result of the evaporation of mixing water. As the hydration is also slower, more water can evaporate. When the evaporation rate of the water is faster than the hydration rate to fill the pores, a higher porosity will be obtained. Additionally, the reduction in available water will slow down the hydration of the GGBS, potentially leading to hindrances. When the hydration stops, the filling effect is not possible anymore. These two factors could result in a higher porosity. M2 contains a lower quantity of GGBS than REF, which explains the difference in the effect of the curing condition. In the case of pure PC binder, the hydration kinetics are faster and will be less affected by the evaporation after the first few hours. Therefore, the same porosity under different curing conditions is observed. Similar observations have been seen by Ortega et al. [45] and Younsi et al. [14]. 

### 4.3. Water Absorption

The water absorption of concrete can be divided into two stages: a rapid and a slow absorption stage [46]. The driving mechanism in each stage is different. The rapid stage is known as capillary absorption. Due to the adhesion forces and surface tension of the concrete, water will flow into the capillary pores. This creates a waterfront within the dry concrete. However, after a few hours, the capillary action will decrease and the water profile becomes stable. At this point, a slow water absorption stage begins. During this stage, water that is in the full capillary pores will move towards gel and interlayer pores. During this stage, water movement is diffusion-driven, which is much slower than under capillary action.

The ingress of water into the printed concrete can be divided into an initial and secondary sorptivity. The initial sorptivity of the rapid stage (according to the ASTM C1585 [47] within the first 6 h) can serve as an indicator of the durability performance [46]. A high initial sorptivity is a strong indication of a coarser pore structure and permeable concrete [48]. The secondary sorptivity (according to the ASTM C1585 [47] measured between 1 and 8 days) shows the ability of the concrete to release air when the voids are filled [49]. A high secondary sorptivity indicates a high quantity of air voids and cracks [50,51]. The parameter can be used to indicate the service life of the concrete [52]. 

**Figure 12:**
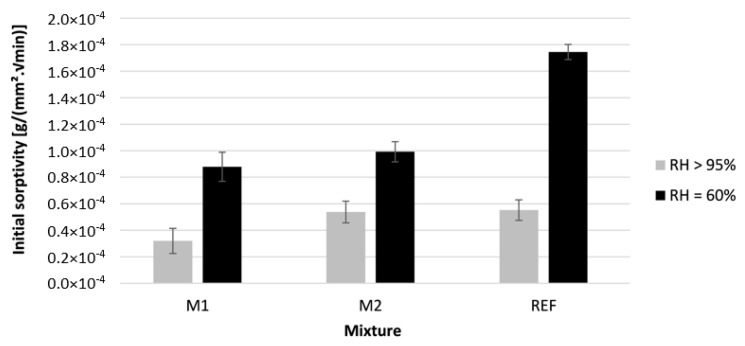
The initial sorptivity measured on 3D printed concrete specimens cured under different curing conditions; n = 3.

**Figure 13:**
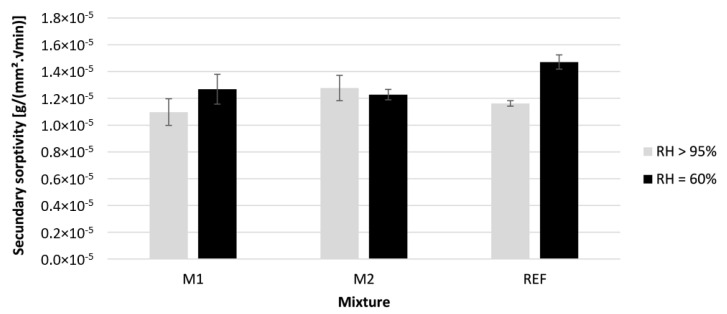
The secondary sorptivity measured on 3D printed concrete specimens cured under different curing conditions; n = 3.

Figure 12 and Figure 13 show the initial and secondary sorptivity of the printed specimens cured under two different ambient humidities. The sorptivity was calculated based on the three tested specimens. The error bars indicate the standard error. 

A significant effect of the curing condition on the initial sorptivity is visible. In the case of RH = 60%, the initial sorptivity increases with the increase of GGBS present in the mixture. This is in line with the results of the open porosity and is therefore not unexpected. The increase in open porosity will lead to a better penetration of water into the concrete. The initial sorptivity of specimens cured under RH > 95% is similar. This follows the same trend as the results of the open porosity. As the open porosity is the same, the same ingress of water was expected. 

The results of the secondary sorptivity are surprising. The increase in evaporation of water normally leads to an increase in capillary pores. As all these pores are filled during the first stage, the secondary sorptivity would not be affected by the curing condition. This is the case for M1 and M2, but not for REF. Based on these results, the RH = 60% specimens contain a larger quantity of macropores than the RH > 95% specimens. This could indicate that the REF samples contained more drying cracks compared to the other two mixtures. A possible reason for this is the lower sand to binder ratio, which increases the risk of experiencing drying shrinkage cracks. 

### 4.4. Accelerated Carbonation 

#### 4.4.1. Sequence 1: The Effect of the Formwork Curing Conditions 

**Figure 14:**
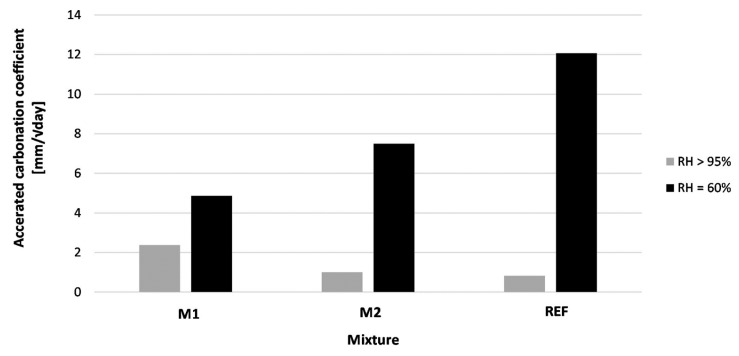
Accelerated carbonation coefficient (concentration = 1%) of 3D printed concrete specimens cured under different curing conditions—n = 3.

Figure 14 shows the accelerated carbonation coefficient of 3D printed concrete specimens cured under different conditions. The carbonation coefficient is obtained by performing a regression analysis on the measured carbonation ingress (n = 3) at different periods. All accelerated carbonation coefficients have a coefficient of determination (R^2^) of at least 0.81. 

The curing conditions have a significant effect on the carbonation resistance of the printed concrete. The magnitude of the influence is dependent on the mixture composition. While for M1 (pure PC as binder) the carbonation coefficient is 2.05 times higher due to improper curing (RH = 60%), this becomes 7.42 and 14.60 times higher for M2 and REF. There are two possible reasons for this effect. First of all, by incorporating GGBS into the concrete mixture, the alkalinity will decrease. The reduction in Ca(OH)_2_ content makes these types of concrete more prone to carbonation. Secondly, in Section 4.2 it was observed that the open porosity is significantly higher. This indicates a higher permeability, which facilitates the ingress of CO_2_. As a result, the carbonation coefficient would be significantly higher than for the M1 mixture. 

In the case of proper curing conditions (RH > 95%), the carbonation resistance of M1 is lower than for M2 and REF. This was unexpected, as a pure PC mixture has a better resistance against carbonations than slag-blended cementitious mixtures. Additionally, the results of Section 4.2 indicate a lower porosity of M1 mixtures compared to M2 and M3. Nevertheless, the difference between the mixtures falls within the measurement error of the performed test, which makes the difference in carbonation resistance between the three mixtures cured at RH > 95% insignificant. 

#### 4.4.2. Sequence 2: The Performance of Properly Cured Elements

**Figure 15:**
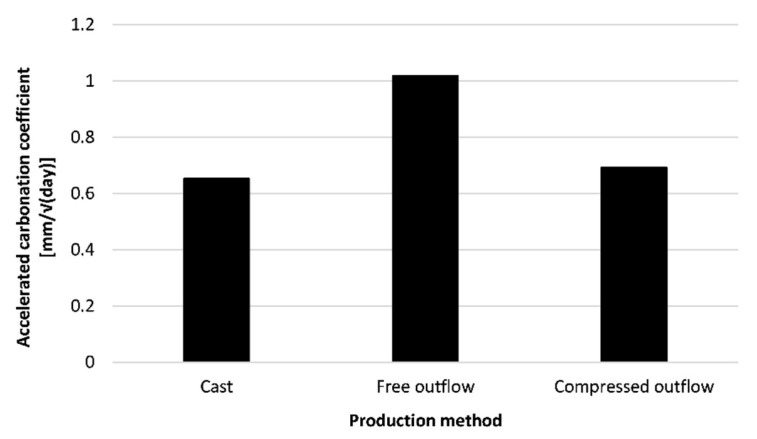
Accelerated carbonation coefficient (concentration = 1%) of 3D printed concrete produced with different methods (REF-mixture cured under RH = 60%).

In Figure 15 (REF-mixture cured under RH = 60%), the accelerated carbonation coefficient for a concentration of 1% CO_2_ is shown. First of all, a significant difference between the results of Figure 14 (REF-mixture) and Figure 15 (Free outflow) is observed. Despite both mixtures being cured in RH = 60%, the specimens for which the results are shown in Figure 14 were not protected against drying in the first day while the specimens for which results are shown in Figure 15 were protected for the first 24 h. This shows that the protection against evaporation during the first day of curing is of major importance. Moreover, the carbonation coefficient becomes similar to the specimens that were cured in RH > 95%. Therefore, protection against evaporation within the first 24 h after printing seems to be crucial. 

Not only the environment, but also the production method plays a significant role. A visible difference can be observed between the casting and printing processes. An explanation can be found in the difference in pore structure and porosity of the printed specimens compared to cast specimens. Printed concrete contains larger pores compared to cast concrete. These larger pores are more interconnected, while in cast concrete this is less common [28]. As a result, it will take more time in printed concrete to see the blocking effect as more and larger pores have to be closed. 

To obtain a construction method closer to casting, side trowels can be mounted on the nozzle. In this case, printed layers could obtain a higher degree of compaction in comparison to layers for which the outflow is free. As the material is better compacted, the pore structure could change, improving the durability aspects. Based on the results shown in Figure 15, a clear difference can be seen between printing with and without side trowels. Printing with side trowels decreases the carbonation ingress. As the material cannot flow freely out of the nozzle, it is compacted which decreases the number of pores in the printed layers. Additionally, the side trowels smoothen the surface, closing possible plastic cracks induced during printing. 

### 4.5. Rapid Chloride Migration 

#### 4.5.1. Sequence 1: The Effect of the Formwork Curing Conditions 

**Figure 16:**
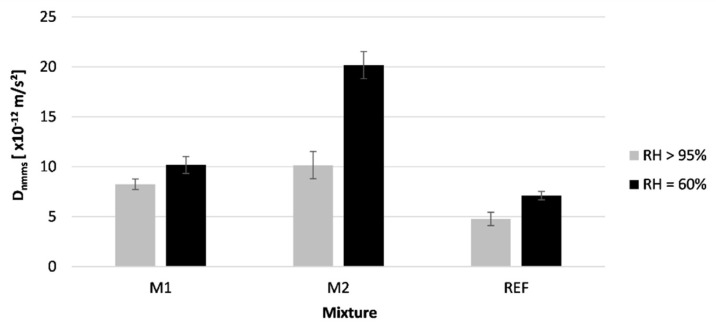
Rapid chloride migration coefficient tested on different mixtures cured in two curing conditions; n = 3.

Figure 16 shows the results of the performed rapid chloride migration test. The error bars indicate the standard deviation of the tested samples. 

The specimens used to define the chloride migration coefficient cured at an RH = 60% showed an increase in chloride migration coefficient for mixture M1, M2, and REF, respectively, with 23.3, 98.9, and 48.9% compared to specimens cured at an RH > 95%. This indicates that the curing conditions have a significant influence on the chloride migration coefficient of the concrete. However, the chloride migration is less affected by the tested mixture, while this was the case for the carbonation resistance and the water absorption. Literature indicates that these latter tests are highly influenced by the quantity of capillary pores present in the mixture. However, in the case of the chloride migration resistance, the pore distribution and size are more important. Moreover, incorporation of GGBS in concrete can improve the chloride binding potential, reducing chloride ingress. Although the chloride binding is expected to be very low during the forced migration test setup, previous research showed that GGBS still reduces the chloride migration coefficient. 

#### 4.5.2. Sequence 2: The Performance of Properly Cured Elements

**Figure 17:**
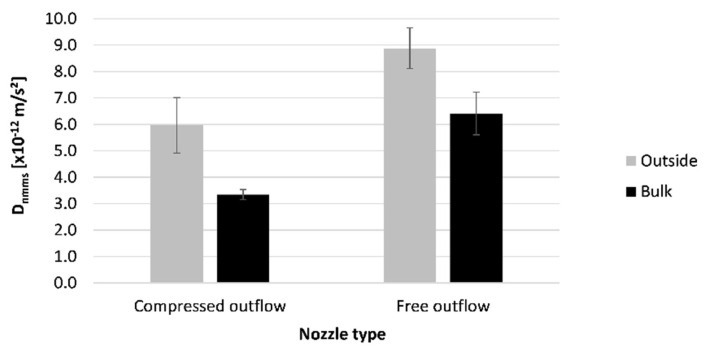
Effect of compressed outflow on the chloride migration coefficient of printed concrete (REF mixture cured under RH = 60%); n = 3.

In Figure 17, the difference in chloride resistance between the surface layer and bulk material is shown for the printed specimens. The error bars indicate the standard deviation of the tested samples. Results show that the outer layer of the printed specimens has less resistance to chloride ingress than the inner material. A decrease of 39–45% in resistance can be noticed. Some plausible causes of this finding are cracks forming during printing and the formation of shrinkage cracks due to immediate exposure of the printed material to the environment [10]. Additionally, the evaporated water during this drying process will reduce the latent hydraulic reaction of slag, leading to a less dense microstructure on the outside of the printed specimens [14]. It was already observed in Section 4.1 that the outer layer of the printed concrete was subjected to a higher level of drying. Therefore, the chloride resistance will differ throughout the thickness of the printed layer. Additionally, the additional compression enforced by the side trowels leads to better compaction and can densify the structure. Therefore, a change in porosity and pore structure can be achieved. 

## 5. Numerical Results: Effects Based on Service Life Analyses

Within this section, the service life of concrete structures with 3D printed formwork is studied in relation to the considered concrete cover thickness and curing conditions. Mind that these reported values are subjected to the limitations of the used model and its parameters. Any changes to the input will alter the outcome. Therefore, the reported results should be seen as indicative values.

### 5.1. Carbonation-Induced Corrosion 

#### 5.1.1. Sequence 1: The Effect of the Formwork Curing Conditions

**Figure 18:**
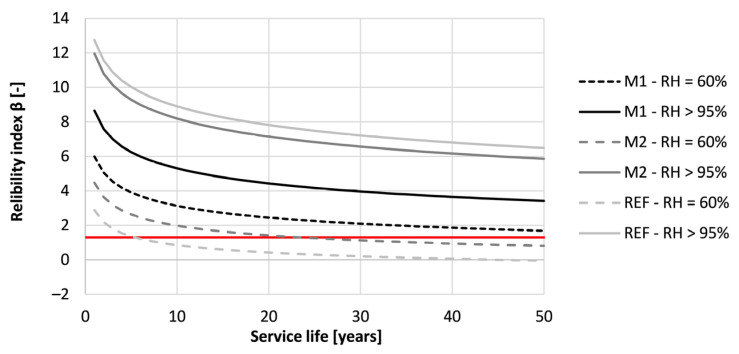
Reliability index of the tested printed concrete specimens (sequence 1) for different exposure times based on prescribed Eurocode 2 design for carbonation-induced corrosion. The red line indicates the required value of 1.3.

Figure 18 indicates the reliability index in time for the tested concrete specimens when designed according to Eurocode 2. The red line indicates the minimum reliability index of 1.3, corresponding to the start of depassivation of the provided reinforcement. 

A significant difference in performance can be observed between the different situations. First of all, all printed concrete that was cured in RH > 95% conditions have a significantly higher reliability index than 1.3 after 50 years of exposure. However, this is not the case for the concrete with the same mix composition cured in RH = 60% conditions. In the latter condition, two out of three tested mixtures (M2 and REF) indicate an earlier depassivation than the anticipated 50 years. According to the results, the service life of REF and M2 designed according to Eurocode 2 is reduced to respectively 5 and 23 years. Therefore, the concrete cover described as 45 mm is not sufficient. 

Additionally, the reliability index of M1 after 50 years increases from 1.69 to 3.41 when changing the curing condition from RH = 60% to RH > 95%. In the case of REF and M2, the reliability index increases respectively from −0.04 to 6.48 and from 0.80 to 5.86. This indicates that the effect of different curing conditions is highly mixture-dependent. Nevertheless, all of the tested mixtures indicate an increased reliability index, indicating a prolonged service life when properly cured (RH > 95). 

**Figure 19:**
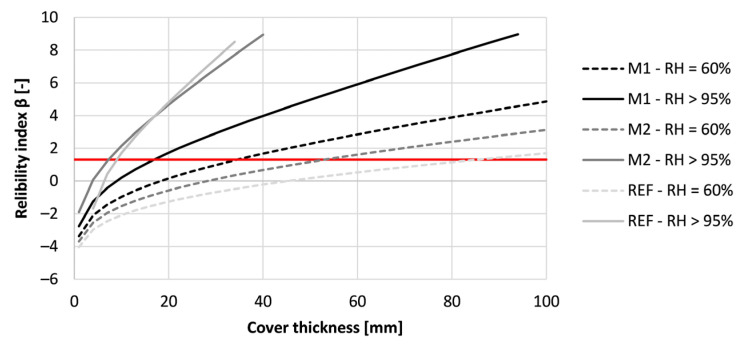
Reliability index of the tested printed concrete specimens (sequence 1) for different cover thicknesses based on a service life of 50 years under carbonation-induced corrosion. The red line indicates the required value of 1.3.

To meet the requirement of a service life of 50 years, the cover thickness could be adapted. The results for the tested concrete mixtures can be seen in Figure 19. As the printed concrete M2 and REF cured at RH = 60% indicated preliminary depassivation when designing according to Eurocode 2, the concrete cover had to be increased. The cover thickness of REF and M2 must be at least 86 mm and 53 mm to ensure a service life of 50 years. M1 under the same curing conditions needs a concrete cover of 35 mm. When the same mixture is cured under RH > 95%, the needed concrete cover for M1, M2, and REF become respectively 17 mm, 8 mm, and 6 mm. Mind that these covers are very low and rather indicative. Nevertheless, the results show that the curing condition of the concrete formwork has a significant influence on the desired thickness of the concrete cover and the structures’ service life. Additionally, the thickness of the concrete could be reduced in comparison to the given design codes when properly cured. 

#### 5.1.2. Sequence 2: The Performance of Properly Cured Elements

**Figure 20:**
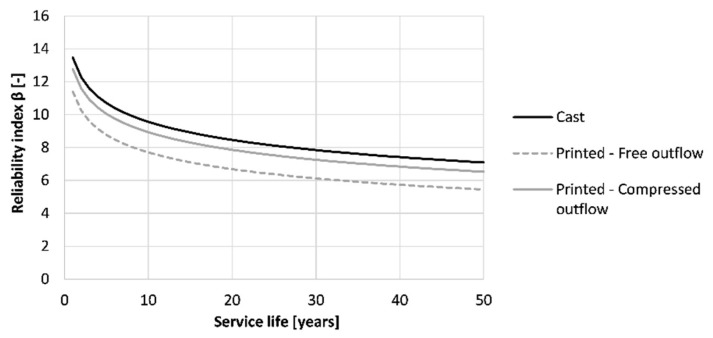
Reliability index of the tested printed concrete specimens (sequence 2) for different exposure times based on prescribed Eurocode 2 design for carbonation-induced corrosion.

Figure 20 shows the effect of the printing method on the service life of the printed structure when being exposed to carbonation corrosion after proper execution. None of the methods seems to induce preliminary failure of the formwork. Casting indicates the best way of producing the formwork (β_50 years_ = 7.09). Printing the concrete and curing it properly results in a reliability index after 50 years of 5.44. However, by adding side trowels to the nozzle, an improvement can be made, which increases the reliability index to a value closer to the one of cast concrete (β_50 years_ = 6.53). 

**Figure 21:**
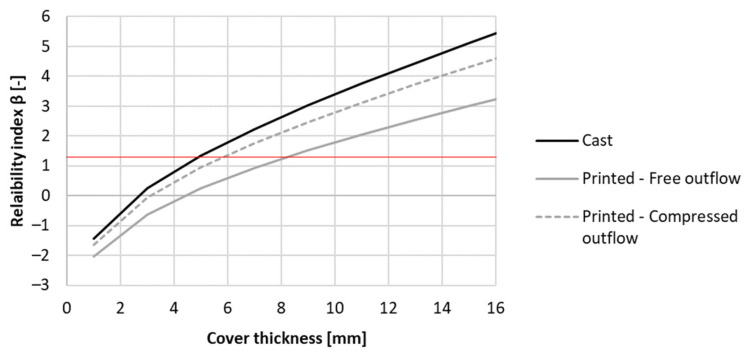
Reliability index of the tested printed concrete specimens (sequence 2) for different cover thicknesses based on a service life of 50 years for carbonation-induced corrosion. The red line indicates the required value of 1.3.

As the resulting reliability index values are high, a reduction of the formwork thickness can be made (see Figure 21). In the case of casting, the required cover thickness is at least 5 mm while for printing with a free outflow this is 9 mm. By adding side trowels to the nozzle, the required thickness is reduced by 3 mm. Despite the improved carbonation resistance by modifying the nozzle with side trowels, the gain in cover thickness is limited. Therefore, the benefits reflected within a real-life application will be low as long as the curing has been performed adequately. At that point, the application of side trowels at the nozzle becomes an aesthetic choice rather than an economical choice.

### 5.2. Chloride-Induced Corrosion 

#### 5.2.1. Sequence 1: The Effect of the Formwork Curing Conditions 

**Figure 22:**
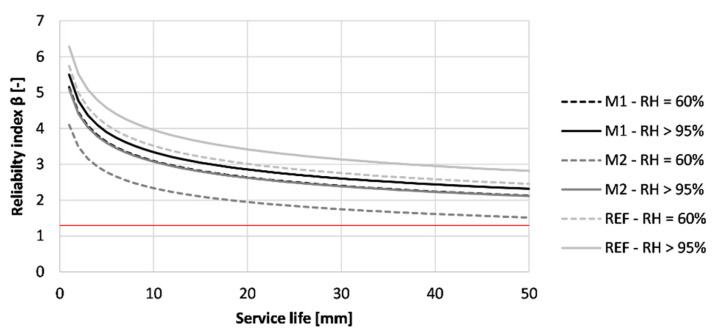
Reliability index of the tested printed concrete specimens (sequence 1) for different exposure times based on prescribed Eurocode 2 design for chloride-induced corrosion. The red line indicates the required value of 1.3.

Figure 22 shows the reliability index for the first 50 years of the tested printed concrete exposed to chlorides. All tested concrete specimens obtained a reliability index that is larger than 1.3 after a service life of 50 years. This means that none of the tested concrete is expected to reach depassivation within the desired life span of the structure. The concrete cured at RH > 95% obtained higher β-values after 50 years (M1: 2.32; M2: 2.12 and REF: 2.82) than concrete cured under RH = 60% (M1: 2.13; M2: 1.52 and REF: 2.46).

**Figure 23:**
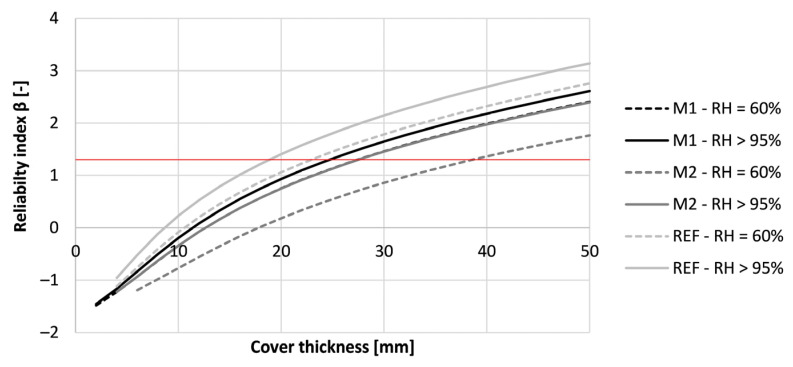
Reliability index of the tested printed concrete specimens (sequence 1) for different cover thicknesses based on a service life of 50 years for chloride-induced corrosion. The red line indicates the required value of 1.3.

Nevertheless, the increase in reliability index by curing the concrete properly is lower for chloride-induced corrosion than for carbonation-induced corrosion. This can be seen in Figure 23. While for carbonation-induced corrosion calculations, the improvement of the curing could lead to a decrease in cover thickness up to 80 mmis is this for chloride-induced corrosion only 11 mm. This indicates a lower effect of the curing on the chloride resistance of the printed concrete. For concrete cured under RH = 60%, the required concrete cover for M1, M2, and REF are respectively 28 mm, 39 mm, and 23 mm. By improving the curing condition to RH > 95% for 28 days, the required concrete cover becomes 25 mm, 28 mm, and 19 mm of these same mixtures. All of the obtained values are lower than the Eurocode 2 prescribed value of 45 mm. Therefore, a proper resistance against chloride-induced corrosion could be expected for these mixtures. 

#### 5.2.2. Sequence 2: The Performance of Properly Cured Elements

**Figure 24:**
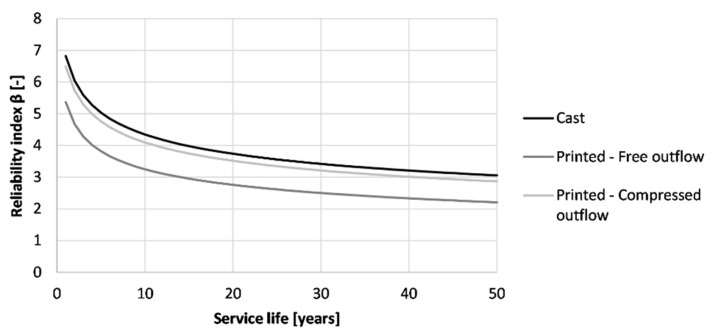
Reliability index of the tested printed concrete specimens (sequence 2) for different exposure times based on the prescribed Eurocode 2 design for chloride-induced corrosion.

Similar to the carbonation resistance, casting can be seen as the best production method for the formwork (β_50 years_ = 3.06), as shown in Figure 24. When printing and properly curing the formwork, the reliability index drops to a value of 2.21. Adding side trowels to the nozzle improves the chloride resistance and increases the reliability index to a value that is nearly similar to the one of the casting procedure (β_50 years_ = 2.87). Nevertheless, taking the thickness of the printed layer equal to the concrete cover described in Eurocode 2, a service life of 50 years could be obtained for the structure regardless of the fabrication method. 

**Figure 25:**
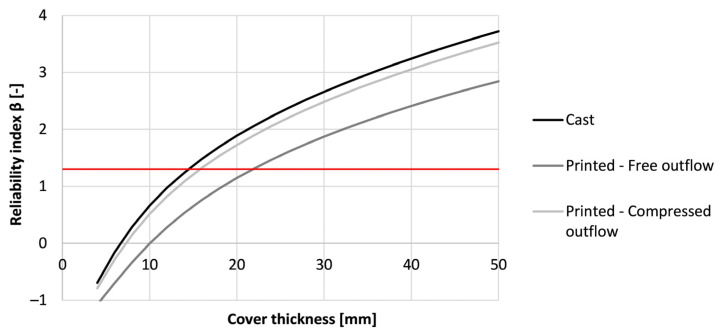
Reliability index under chloride-induced corrosion of the tested printed concrete specimens (sequence 2) for different cover thicknesses, based on a service life of 50 years. The red line indicates the required value of 1.3.

When designing for a service life of 50 years, the concrete cover can be adjusted. Figure 25 shows the minimum concrete cover needed for the printed concrete used with different placement procedures. In the case of casting, a thinner concrete cover (15 mm) can be used in comparison to printing with a free outflow (22 mm). However, by better compacting the concrete during extrusion due to the added side trowels, the concrete cover can be reduced to 16 mm. This indicates that nozzle adjustments can improve the chloride resistance even when an optimal curing of the formwork has been performed. 

### 5.3. Limitations to the Prediction Method

In the previous sections, it has been shown that the formwork is a protective layer against chloride- and carbonation-induced corrosion when properly cured. Despite the promising results, some limitations must be pointed out concerning the design.

The accelerated chloride and carbonation test is limited to printed formwork without cracks. However, early age casting of the infill material leads to cracking of the formwork material within the first 28 days. These cracks will lead to a significant reduction in service life [53]. In case of significant cracking, the formwork should not be taken into account as concrete cover. Therefore, proper action should be taken to prevent cracking of the formwork. Similar actions apply to cracks at the interface between the cast and the printed material [31]. 

While the printed formwork material is considered to be the concrete cover, the reinforcement cannot be placed immediately next to the formwork. To ensure proper bonding between reinforcement and infill concrete, a sufficiently large spacing between rebars and formwork is needed. This spacing is dependent on the maximum aggregate size used in the cast concrete. The additional concrete layer will protect the reinforcement too. As a consequence, the real concrete cover exists out of two concrete types and an interface with different resistance against aggressive substances. This creates a difficulty in designing the needed concrete cover. 

In case of low evaporation protection and a high exposure surface, printed concrete can be more prone to develop a dry affected zone. This area has a lower degree of hydration and a higher porosity compared to the bulk material. Therefore, estimating the service life based on the bulk material will result in an overestimation. Therefore, real printed specimens should be investigated. 

## 6. Conclusions

In this paper, the applicability of 3D printed concrete as formwork material was evaluated. Based on experimental and numerical evaluation, the following conclusions can be drawn:

- Evaporation has a significant effect on the durability of the printed concrete. Early-age drying resulted in an increase in porosity, an increase in water absorption, and a decrease in carbonation resistance. Therefore, evaporation of the mixing water should be prevented;
- The incorporation of GGBS in the 3D printed mixture makes the concrete more prone to the effects of evaporation on the durability parameters such as porosity, chloride ingress, and carbonation resistance;
- Nozzle adjustments such as side trowels can improve the durability of printed structures. The carbonation and chloride resistance of the printed concrete compared to a free outflow are increased;
- The 3D printed formwork is suitable as a concrete cover when correctly applied. In the performed analysis, properly cured (RH > 95%) printed formwork was suitable when designed according to Eurocode 2, whereas improperly cured (RH = 60%) printed formwork was rarely applicable. Therefore, special measurements have to be taken during construction to ensure proper curing in a way that the required service life is obtained.

## Figures and Tables

**Table 1:** Mix compositions and properties for the used printable concrete.

	Unit	REF	M1	M2
Sea sand (0/1)	Kg/m^3^	943	1222	1222
CEM I 52.5 N (MF)	Kg/m^3^	472	815	611
GGBS	Kg/m^3^	472	0	204
Water	Kg/m^3^	330	285	285
Superplasticizer	Kg/m^3^	2.1	4.2	3.2
VMA	Kg/m^3^	0.9	0.8	0.8
Density	Kg/m^3^	2220	2327	2326
Water-to-binder ratio	-	0.35	0.35	0.35
Sand-to-binder ratio	-	1	1.5	1.5

**Table 2:** Mix compositions and properties for the used infill concrete.

	Unit	SCC
Rhine sand (0/4)	Kg/m^3^	853
Rhine gravel (2/8)	Kg/m^3^	263
Rhine gravel (8/16)	Kg/m^3^	434
Limestone	Kg/m^3^	240
CEM I 52.5 N (MF)	Kg/m^3^	360
Water	Kg/m^3^	165
Superplasticizer	Kg/m^3^	3.2
Density	Kg/m^3^	2318
Water-to-binder ratio	-	0.46

**Table 3:** Overview of the performed tests in sequence 1 with the corresponding mixtures, curing conditions, test dates, and the samples tested for each combination.

Test	Curing Condition	Mixtures	Testing Dates	Samples/Testing Date
Evaporation of water	RH = 60, RH > 95	REF	0, 1, 2, 4 days after mixing	3
Porosity based on vacuum saturation	RH = 60, RH > 95	REF, M1, M2	28 days after printing	3
Water absorption	RH = 60, RH > 95	REF, M1, M2	28 days after printing	3
Accelerated carbonation	RH = 60, RH > 95	REF, M1, M2	0, 1,7, 14, 28 after printing	3
Rapid chloride migration test	RH = 60, RH > 95	REF, M1, M2	28 days after printing	3

**Table 4:** Overview of the performed tests in sequence 2 with the corresponding production method, test dates, and the samples tested for each combination.

Test	Production Method	Testing Dates	Samples/Testing Date
Accelerated carbonation	Printed with free outlet, printed without free outlet, cast	64, 71, 78, 92, 120 days after printing	1
Rapid chloride migration test	Printed with free outlet, printed without free outlet, cast	64 days after printing	3

**Table 5:** Parameters for service life estimation: carbonation-induced corrosion (model parameters and distributions adapted from [7], weather-related data from [40,41]).

Parameter		Distribution	Values
Relative humidity real life	${RH}_{real}$	Beta	BetaD (75/9/40/100)
Relative humidity reference	${RH}_{ref}$	Constant	65
Exponent	$f_{c}$	Constant	5.0
Exponent	$g_{c}$	Constant	2.5
Period of curing	$t_{c}$	Constant	Sequence-dependent
Exponent of regression	$b_{c}$	Normal	ND (−0.567/0.024)
Natural CO_2_ concentration	$C_{s}$	Normal	ND (0.00082/0.0001)
Time reference	$t_{0}$	Constant	0.153
Carbonation ingress after 28 days of exposure	$x_{c}$	Normal	Test data
Time constant	τ	Constant	420
Regression parameter	$k_{t}$	Normal	ND (1.25/0.35)
Error term	$\varepsilon_{t}$	Normal	ND (315.5/48)
Probability of driving rain	$p_{sr}$	Constant	0.5
Exponent of regression	$b_{w}$	Normal	ND (0.446/0.163)
Time of wetness	TOW	Constant	ND (0.33/0.05)
Concrete cover	*d*	Lognormal	LOGND (45/6)

**Table 6:** Parameters for service life estimation: chloride-induced corrosion (model parameters and distributions adapted from [9], weather-related data from [40,41]).

Parameter		Distribution	Values
Chloride migration coefficient	D_RCM_ (t_0_)	Normal	Based on results
Aging coefficient	a	Beta	BetaD (0.65/0.12/0/1.0)
Testing date RCM	t_0_	Constant	0.175
Temperature coefficient	$b_{e}$	Normal	ND (4800/700)
Ambient air temperature	$T_{real}$	Normal	ND (283/6.2)
Reference temperature	$T_{ref}$	Constant	293
Chloride content at a certain time at depth d	$C_{s}$	Lognormal	LND (1.5/1.45)
Initial chloride content	$C_{0}$	Constant	0
Critical chloride content	$C_{crit}$	Beta	BetaD (0.60/0.15/0.2/2)
Convection zone	∆x	Constant	0
Concrete cover	*d*	Normal	ND (45/6)

## Data Availability

The data presented in this study are available on request from the corresponding author.
